# Significance of Notch Signaling in Salivary Gland Development and Diseases

**DOI:** 10.3390/jcm14103325

**Published:** 2025-05-10

**Authors:** Margherita Sisto, Sabrina Lisi

**Affiliations:** Department of Translational Biomedicine and Neuroscience (DiBraiN), Section of Human Anatomy and Histology, University of Bari “Aldo Moro”, 70123 Bari, Italy; sabrina.lisi@uniba.it

**Keywords:** Notch, salivary gland, development, Sjögren’s syndrome, adenoid cystic carcinoma

## Abstract

Notch-mediated signaling pathways represent a system that is conserved from an evolutionary point of view, demonstrating a key role in determining cell fate in development; in fact, Notch operates at multiple levels during tissue and organ organization, intervening in the key processes of organogenesis. As a consequence of this, a dysregulation of the Notch-mediated pathways leads to the onset of various pathological conditions such as autoimmune diseases or tumors. The activation of Notch-mediated molecular pathways has also been demonstrated in the development of salivary glands (SGs) and in associated pathologies. Although the numerous advances made in recent years have clarified various aspects of the activation of transductional cascades involving Notch in SGs development and diseases, there are still many aspects that require experimental investigation. In this review, we report, for therapeutic purposes, what is present in the literature relating to the mechanisms regulating the development of Notch-mediated SGs and the most recent discoveries relating to SGs pathologies that derive from alterations of the Notch-mediated pathways.

## 1. Introduction

The Notch pathway is a highly preserved signaling system that plays multifaceted roles in a plethora of molecular events, such as cell proliferation, cell death, EMT (epithelial–mesenchymal transition), tumor aggressiveness, and cell fate determination [1,2]. It is also implicated in the reorganization and transformation of tissues and in intricate processes with other signaling pathways. The trigger of Notch signaling occurs through a tightly regulated cascade of proteolytic cleavages with subsequent activation of downstream target genes that influence and control these essential cellular processes [3,4]. Not surprisingly, aberrant Notch signaling is linked to the pathogenesis of numerous human diseases, including cancer and autoimmune diseases [5]. Although many recent reports have highlighted various aspects of the Notch signaling mechanism and its intricate orchestration, there is still much to understand about how the Notch signaling pathway exerts complex regulatory mechanisms in so many developmental and regenerative processes [6,7,8]. Salivary gland (SG) development involves the interactions of epithelial, mesenchymal, endothelial, and neuronal cells, and it is established that several molecular pathways cooperate to determine the correct architecture of the glandular tissue. Currently, our knowledge of the mechanisms by which Notch signaling pathways interfere with SGs cell organization in the tissue architecture and how Notch could function during SGs formation is limited. In this review, we provide an overview of the mechanism and regulation of the Notch signaling pathway in several aspects of SGs development. We will highlight progress in understanding how cell fate decisions are strictly linked with branching morphogenesis and the formation of the arborized ductal tree in SGs. In addition, we will highlight the latest findings demonstrating an involvement of Notch signaling in autoimmune and non-autoimmune diseases of SGs. Finally, we will summarize developments in the therapeutic targeting of Notch signaling and the advantages and disadvantages of this approach for treating SGs pathologies.

## 2. Notch Signal Transduction System

The Notch gene encodes a transmembrane receptor that triggers the Notch signaling pathway, a cascade highly preserved throughout evolution that coordinates several physiological processes through network communication [3]. Consequently, a misregulation of Notch signaling leads to a wide range of human disorders, such as developmental syndromes and adult-onset tumors [5]. Notch family proteins (Notch 1, 2, 3, and 4) are heterodimers of 300 kDa with more than 2500 amino acids and three main portions: Notch extracellular domain (NECD), transmembrane domain (TMD), and Notch intracellular domain (NICD) [9]. The NECD consists of different epidermal growth factor (EGF)-like repeats and a negative regulatory region (NRR), restricted by O-glycans to regulate the Notch receptor’s affinity for multiple ligand proteins [10,11].

**Figure 1:**
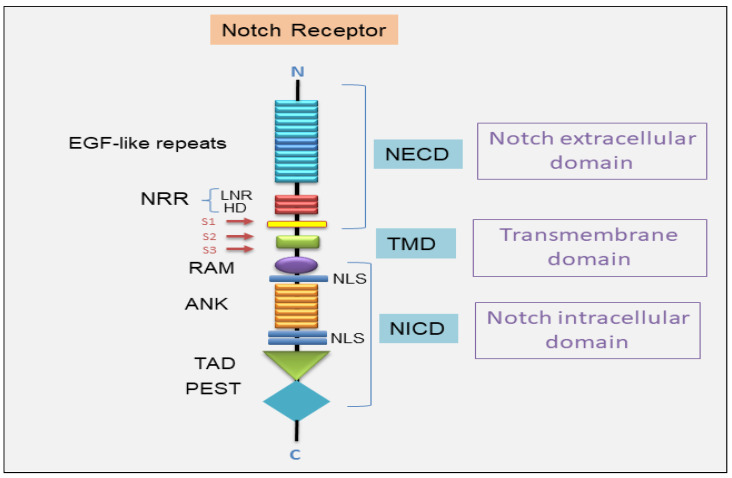
A schematic representation of the structure of the Notch receptor. The Notch extracellular domain (NECD) includes the EGF-like repeats, followed by three Lin-12/Notch repeats (LNR) and the heterodimerization domain (HD). The LNR segments and the HD constitute the negative regulatory region (NRR). The transmembrane domain (TMD) follows. The Notch intracellular domain includes the RBPJ association module (RAM), followed by ankyrin (ANK) repeats, the transcriptional activation domain (TAD), and the C-terminal terminal proline/glutamic acid/serine/threonine-rich motifs (PEST) domain. Nuclear localization sequences (NLSs) are pinpointed on both sides of the ANK domains. S1/S2/S3 are cleavage sites for activation of Notch receptor. DLL (Delta-like); JAG (Jagged).

Notch1 and Notch2 are highly similar proteins, while Notch3 and Notch4 are more different. Notch receptors reach out with five ligands (Delta-like 1, 3, 4, and Jagged-1, 2) [12,13]. All Notch receptors are thought to be induced by the same process involving juxtacrine interactions between adjacent cells when a Notch ligand on one cell is presented to a Notch receptor on a neighboring cell [5] (Figure 1). There are two mechanisms by which Notch can modulate cellular differentiation. They are the canonical and non-canonical pathways [14,15]. Both canonical and non-canonical pathways are typically triggered by the linking to Delta-like or Jagged ligands at the EGF region of the Notch-1 extracellular domain [9]. This binding starts a sequence of events that ultimately leads to transcriptional induction of Notch target genes in the receiver cell [3,16,17,18]. In signal-sending cells, Notch ligands bind to Notch receptors on signal-receiving cells. The ligands, through a mechanism that involves clathrin-mediated endocytosis (CME), produce a pulling force for the binding receptors [19]. Notch ligand endocytosis requires the ubiquitylation by Mib1 (or other E3 ubiquitin ligases), typically followed by the recruitment of the clathrin adaptors epsin 1 and/or epsin 2. The force produced by the CME of the ligand prompts on the Notch receptor, leading to the exposure of a cleavage site at the NRR located next to the TM domain [18,20].

### 2.1. The Canonical Notch Signaling Pathway

**Figure 2:**
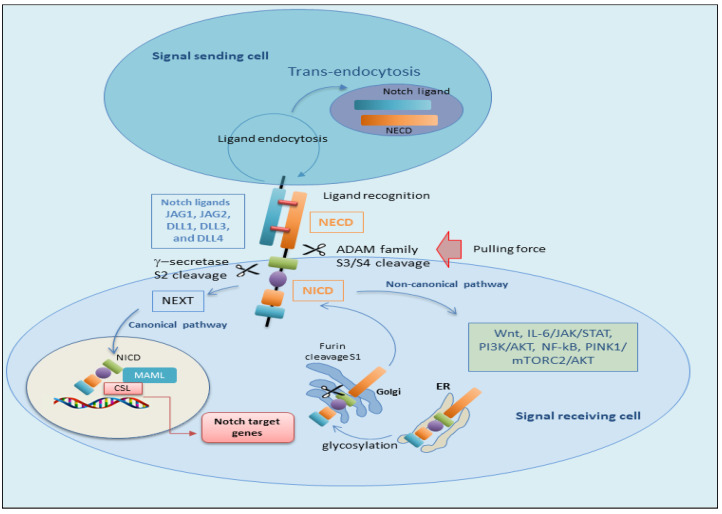
Overview of the Notch signaling pathway. Canonical Notch signaling pathway. Notch receptors are synthesized as single-chain precursors, transported and glycosylated at the EGF-like repeat domain in the endoplasmic reticulum, and undergo S1 cleavage in the Golgi by furin into an extracellular and a transmembrane subunit. Then, the matured proteins are transported and transferred in the plasma membrane. Interaction between Notch receptors and their ligands creates a pulling force that allows ADAM proteases to remove the extracellular subunit and the γ-secretase complex to cleave the receptor (at the S2 and S3 cleave site, respectively), releasing the intracellular domain (NICD) to the cytoplasm of the signal-receiving cell. After S2 cleavage, the remaining part of the Notch receptor is called NEXT. NEXT is further cleaved at the S3 site, releasing NICD or transported into lysosomes for degradation. Concurrently, the extracellular segment of the Notch (NECD) and the ligand are internalized by the signal-sending cell. In signal-sending cells, Notch ligands are distributed on the cell membrane and can bind to Notch receptors on signal-receiving cells. However, the ligands are inactive before ubiquitylation. After ubiquitylation, ligands can be endocytosed, thus producing a pulling force for the binding receptors. The second proteolytic cleavage generates the translocation of the NICD into the nucleus. In the nucleus, NICD binds with a transcription factor, RBP-Jκ (also known as CSL, coactivator Mastermind-like (MAML) proteins), and constitutes an activated transcriptional complex. Then, the activated complex upregulates the expression of target genes. Non-canonical pathway. Non-canonical signaling pathways are able to trigger signaling independently by interaction of CSL. NICD can interact with the Wnt, IL-6/JAK/STAT, PI3K/AKT, NF-kB, PINK1/mTORC2/AKT pathways at the cytoplasmic and/or nuclear level to regulate the transcription of target genes. ADAM (A Disintegrin And Metalloproteinase); AKT (AKT Serine/Threonine Kinase); IL-6 (Interleukin-6); JAK (Janus Activated Kinase); mTORC2 (mammalian target of rapamycin complex 1); NF-kB (Nuclear factor kappa B); NEXT (Notch extracellular truncation); PINK1 (PTEN-induced putative kinase); PI3K (phosphoinositide 3-kinase/protein kinase B); Wnt (wingless-type).

The canonical Notch signaling pathway implicates a set of complex phases that regard the progression and trigger of Notch proteins. Initially, Notch proteins are transferred to the endoplasmic reticulum as single-stranded precursors. Within the endoplasmic reticulum, EGF repeats of the Notch receptor are converted by the addition of O-glycans [21,22]. When the Notch receptor is glycosylated, it is transported to the Golgi apparatus, and a furin-like convertase cleaves the S1 site in the extracellular portion of the Notch transmembrane region, leading to the formation of two specific portions: the NECD and the TMD [23]. These fragments subsequently unite through a Ca^2+^ dependent non-covalent bond, producing the mature form of the Notch receptor as a heterodimer. The mature Notch receptor is then transferred to the cell surface. At this time, the Notch heterodimeric transmembrane receptor links to the Notch transmembrane ligand localized on neighboring cells [20]. Subsequently, the S2 cleavage site of the Notch receptor is then cleaved by molecules of the ADAM metalloproteinase family, in particular ADAM10 or ADAM17 [24,25]. After S2 cleavage, the process determines the formation of a temporary extracellular peptide named ‘NeXT’ (Notch extracellular truncation), which consists of the TMD and NICD. The TMD comprises an extracellular short segment and stored cysteine residues forming heterodimers [26]. The NICD contains an intracellular RBP-J-associated molecule (RAM), seven ankyrin repeat (ANK) domains, and tandem nuclear sequences (NLSs) that are located on each side of the ANK domain. At the end of the intracellular domain (C-terminus), there are preserved proline/glutamic acid/serine/threonine-rich motifs (PEST) domains that include degradation signals, crucial for the stability of the NICD [27]. Therefore, NEXT becomes a substrate for γ-secretase at the S3 cleavage site by γ-secretase or is endocytosed into endosomes [20]. Thus, γ-secretase cleaves NEXT and leads to the release of the soluble NICD, free to translocate into the nucleus where its RAM domain initially interacts with the transcription factor CBF1/suppressor of hairless/Lag1 (CSL, also named RBPJ). This synergy between NICD and CSL leads to the constitution of a co-activator acknowledged as mammalian mastermind-like 1–3 (MAML1–3) proteins. This assembly results in the formation of a co-activator complex leading to the upregulation of the transcription of downstream Notch target genes. Interestingly, in the absence of NICD binding, CSL recruits co-repressor proteins to downregulate the expression of target genes [28]. A schematic representation of the canonical Notch-mediated pathway is reported in Figure 2.

### 2.2. The Non-Canonical Notch Signaling Pathway

While canonical Notch signaling is well known to play an active role in several steps during development as well as in multiple cell fate decisions, in the last few years, investigations have identified a novel non-canonical role for Notch in which RBPJκ/CSL are not required in the activation of Notch signaling [29,30,31]. Interestingly, accumulating evidence has demonstrated that many processes of non-canonical signaling are linked to pathological disorders such as cancer and to the aberrant activation of the immune system, while many physiological signals require the canonical Notch pathway. Indeed, the inhibition of non-canonical Notch signaling may provide the possibility to block some pathological conditions, leaving many other normal physiological processes intact [6,30]. Notch signaling is also known to interact with other signaling pathways that involve downstream targets and control cellular functions. Recent studies in vertebrates and invertebrates revealed that Notch can also influence the expression of related genes independent of RBPJ, in a non-canonical fashion [32]. It has been reported that the non-canonical Notch signaling may be triggered by ligand-independent mechanisms and might not necessitate the Notch receptor cleavage. Therefore, the non-canonical Notch signaling pathway may regulate angiogenesis, breast cancer, and cells differentiated in the initial phase [20,33]. Notably, the most well-investigated and maintained events of non-canonical Notch function are the regulation of Wnt/β-catenin signaling [34], the Janus kinase/signal transducer and activator of transcription (JAK/STAT) pathway [35], the phosphoinositide 3-kinase/protein kinase B (PI3K/AKT) pathway [36], and the NF-κB pathway at the post-translational level. In epithelial cells derived from the human breast, Notch-activated expression of Wnt signaling receptor FZD7 is needed for non-canonical Notch3 signaling [37]. A very recent study identifies IL-6 as a target gene in the non-canonical Notch activation pathway in breast cancer cells; in fact, the activation of the IL-6/JAK/STAT molecular axis in breast cancer cells has been shown to be mediated by IKKα/IKKβ of the NF-κB signaling cascade [38,39]. In this regard, it is significant to note that Wang et al. reported that extracellular vesicles contain active Notch receptors and regulate the non-canonical Notch signaling cascade. These vesicles sprout directly on the cellular membrane and can be transferred to recipient cells to mediate specific Notch signaling and may have important implications in the invasive phenotype of breast cancer [40]. Authors have discovered that non-classical Notch signaling interacted with PTEN-induced kinase 1 (PINK1) to modulate mitochondrial function and induce mammalian target of rapamycin complex 2 (mTORC2)/AKT signaling, which preserved brain tumor-forming stem cells [41]. Perumalsamy and colleagues evidenced a novel non-classical Notch signaling cascade independent of RBPJ, where NICD cooperates with the mTOR-Rictor complex, leading to the activation of AKT/PKB that blocks apoptosis triggered by cytokine removal in human cells, influencing cell survival [42]. However, it was demonstrated that Notch3 has a pro-apoptotic role in regulating tumor angiogenesis independently of the Notch canonical pathway. The Notch 3 ligand Jagged-1, produced by tumor cells, is upregulated in human lung cancer-associated endothelial cells and inhibits the apoptosis induced by the altered Notch3 expression in tumor vasculature. Consequently, using Notch3 mutant mice, it was shown that tumor progression and angiogenesis are augmented when Notch3 is silenced [43,44]. These non-classical mechanisms allow evolutionarily conserved Notch signaling to carry out more specific functions and may uncover new therapeutic targets as additional mechanisms are revealed in cancers. Figure 2 reports a schematic comparison between the canonical and non-canonical pathways of Notch activation.

## 3. Notch Signaling in Salivary Gland Development

**Figure 3:**
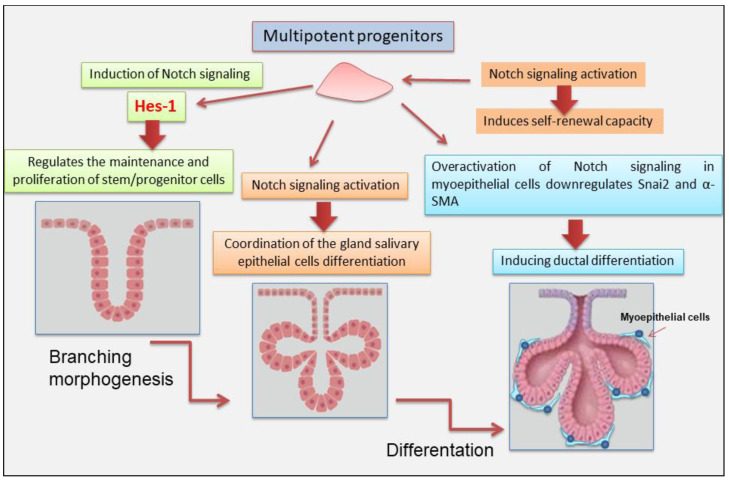
Schematic representation of the Notch signaling in salivary gland branching morphogenesis. Notch signaling is strongly implicated in the regulation of the maintenance, proliferation of stem/progenitor cells and in the coordination of the cell differentiation. Hes (hairy/enhancer of split); α-SMA (alpha-smooth muscle actin).

SGs development implies a mechanism of branching morphogenesis that proceeds via tip-driven regulation of a precise cellular differentiation into acinar, myoepithelial, and ductal (basal and luminal) sub-lineages. Various molecules were identified for SGs morphogenesis that are involved with gland differentiation and development. An important molecule in insects’ SGs differentiation is Notch, which was first identified in fruit flies. In Drosophila Notch-gene mutants [45], SGs do not develop [46]. Over the past few decades, Notch was investigated in various organs, and it has been recognized as a master regulator of cell fate decisions, and it is highly conserved as a trigger of signaling pathways. Therefore, it is involved in the coordination between neighboring cells during developmental tissue processes [3,16,47]. Notch signaling influences the expression of luminal lineage in lacrimal glands. Inhibition of this signaling blocks the maturation and maintenance of the lacrimal glands, demonstrating the dependence on Notch signaling [48]. Therefore, induction of Notch signaling was also demonstrated to regulate branching morphogenesis of the mammary ductal tree. In particular, Notch 2 influences myoepithelial cell lineages and differentiation hierarchy [49]. In the last decade, important studies were conducted on SGs tumors, demonstrating the activation of Notch signaling in salivary adenoid cystic carcinomas (AdCCs); however, little is known about Notch signaling during human SGs differentiation. One interesting approach to understanding the intricate cell–cell communication in SGs morphogenesis was the use of the human SGs cell line (HSG) as an experimental model to investigate the role of Notch signaling in the gland differentiation and development. Dang et al., 2009 have shown that the HSG cell line, as well as human and rat SGs, expresses all four Notch receptors and many of its cognate ligands [50]. Upon HSG differentiation to acinar-like features, the Notch pathway is activated, inducing Hes-1 expression that regulates the maintenance and proliferation of stem/progenitor cells as an essential driver of the Notch signaling pathway. Furthermore, HSG differentiation is inhibited by γ-secretase inhibitors and siRNA-mediated silencing of all four Notch receptors, indicating that Notch signaling is strongly implicated in the coordination of the HSG differentiation [50]. Chatzeli et al. have investigated the functional role of Notch on SGs development, treating SG explants of mice at stage E14.5 with *N*-[*N*-(3,5-Difluorophenacetyl)-Lalanyl]-S-phenylglycine t-butyl ester (DAPT), an inhibitor of Notch signaling. Interestingly, after 2-day treatment, a severely reduced branching compared with controls was observed, and the explants display larger mesenchymal space, indicative of a reduced density of epithelial cells [51]. Growing evidence has revealed that a sophisticated crosstalk between Notch and Snai2 exists, which initially has been investigated in cancer process, evolution of endothelial cells, and neural crest [52]. Indeed, recently, authors have focused their research on myoepithelial cells of the SGs that exhibit both epithelial and mesenchymal features. This interesting study demonstrated that the excessive activation of Notch signaling in myoepithelial cells downregulates Snai2 and α-SMA, triggering ductal differentiation in the cellular culture, whereas inhibition of Notch signaling by DAPT showed an increased expression of Snai2 in the myoepithelial cell cultures. This suggests that the loss of Snai2 in ME cells might strongly induce epithelial properties because Snai2 activation inhibits E-cadherin expression via epigenetic modification and Notch cooperates to orchestrate these mechanisms [52]. Recently, an important report has investigated the novel role of Notch signaling in modulating the regenerative capacity of SGs stem/progenitor populations undergoing radiation-induced DNA damage [7]. To understand if surviving stem/progenitor cells depend on Notch signaling, mouse submandibular SGs organoids (mSGOs) were irradiated and treated with the Notch inhibitor. Interestingly, inhibitory treatment impaired the self-renewal capacity of irradiated mSGOs. Additionally, Notch inhibition significantly impaired the migration abilities of irradiated SGs cells, thus highlighting the critical role of Notch signaling in sustaining both stemness and migratory potential in irradiated SGs tissue. This report revealed a crucial role of Notch signaling in maintaining adult SGs stem/progenitor cell function in regenerative conditions [7]. Moreover, further investigations are necessary to elucidate the precise mechanism and effects of activating or inactivating the Notch pathway on cell fate. More recently, Lu et al. 2025 [53] have used organoid culture composed of salivary acinar, ductal, and myoepithelial cells using hMSGs obtained from healthy individuals. These organoids conserved the same structural heterogeneity and specific secretory activity as actual human minor salivary glands (hMSGs) [53]. The addition of DAPT effectively stimulates the maturation of hMSG organoids in vitro, demonstrating as Notch inhibition promotes the differentiation of organoids. These results are consistent with those observed in the major SGs. In other glandular tissues such as breast, pancreatic, and liver, the activation of the Notch pathway was also shown to promote the activity of stem/progenitor cells [53]. However, in spite of several studies, it also remains uncertain whether the processes observed in organoids accurately reflect those in vivo. Figure 3 illustrates recent findings that support the role Notch plays in SGs development.

## 4. The Role of Notch in the Autoimmune Disease Sjögren’s Syndrome

Patients affected by primary Sjögren’s syndrome disease (pSjD) have as their primary characteristic the altered tissue organization and consequently a reduced functionality of the exocrine glands, which manifests itself primarily in the SGs and lacrimal glands; these types of glands undergo an autoimmune attack in pSjD patients, which causes the inflammatory state to become chronic. Over the last decade, an increased correlation has been demonstrated between the diagnosis of pSjD and the risk of developing diffuse large B-cell lymphoma (DLBCL) and marginal zone lymphoma; in particular, recent clinical studies have highlighted a close association between pSjD and the concomitant onset of the parotid MALT lymphoma [54]. The situation just described in pSjD supports a widespread awareness among scholars of the presence of a close association between the chronic inflammation that characterizes, for example, autoimmune diseases and a greater risk of developing tumors of the immune system, such as specific lymphomas. And, in fact, pSjD is now included in the list of diseases with a high risk of evolving towards the onset of lymphomas [54,55]. Although, as has been known for decades, all autoimmune diseases can be characterized by an increased risk of contracting lymphomas, some of these pathologies, including pSjD, have a greater risk. Currently, it is necessary to deepen and clarify the role played by Notch in pSjD; recent research has demonstrated an increase in Notch2 mRNA expression in B cells located in the marginal zone of the SGs; Notch mRNA was also detected in the germinal centers of tonsil biopsies from patients with pSjD [56]. Elevated Notch2 and PR domain zinc finger protein 1 mRNA levels and increased B-lymphocyte maturation-induced maturation protein 1 (BLIMP-1) mRNA expression were demonstrated within clusters of transient type II B cells present in pSjD SGs [57]. For greater clarity, let us remember that the pro-B cells deriving from the bone marrow, due to gene activation, transform into pre-B cells. After leaving the bone marrow, they transform in the spleen into transient B lymphocytes of type 1 B cells and type 2 B cells. Type 1 B cells show high levels of IgM, CD10, and CD32 and low expression of IgD and CD21. Upon encounter with their cognate Ag, type 1 B cells differentiate into type 2 B cells characterized by moderate expression of IgM, IgD, CD10, and CD32 and low expression of CD21. Circulating B lymphocytes originate from these cells and organize themselves in the lymphoid follicles as germinal centers (GCs) or leave the bloodstream and colonize the splenic marginal zone (MZ) [58]. It is well established that transitional type-2 B cells are expanded in the ectopic GC of SGs in pSjD patients [56], where they could drive the inflammatory evolution of the disease towards a chronic condition, precisely through pathways that involve the activation of Notch/BLIMP-1 [56,57]. Marginal zone lymphoma (MALT lymphoma) of the parotid gland represents a small subgroup of lymphomas of the head and neck (NHL). Currently, a diagnosis of pSjD leads to evaluating or considering a greatly increased risk of MALT of the parotid gland [59]. A clear correlation between MALT and Notch has been demonstrated in MALT lymphomas, demonstrating alterations in genes of the Notch pathway. Notch2, a key regulator of marginal zone B cell development, was identified as the most frequently mutated gene, and, in addition, genetic lesions in the transcription regulator SPEN (Split Ends), in the positive regulator DELTEX1, and in Notch1 were demonstrated [60]. Mutations in members of the NF-κB pathway have also been implicated in MALT lymphoma [61], such as mutations of TNFAIP3 (A20) [62,63]. Therefore, once again, a correlation between pSjD, lymphoma susceptibility, and Notch activation seems to be conceivable [64]. Obviously, it is not yet clear how the possibly canonical and non-canonical pathways mediated by Notch can drive the development of MALT lymphomas associated with pSjD. Further deductions regarding an involvement of Notch signaling in the pathogenesis of pSjD derive from the evidence, reported in the first part of this review, of an involvement of Notch in the development of SGs, a process that appears to be compromised or defective in pSjD patients [65]. In pSjD, several recent experiments have demonstrated altered expression of molecular components of the signal transduction pathway involving WNT. This influences cell fate during the embryonic development of SGs. Wnt1 and Wnt3 show high levels of expression in pSjD SGs, and, furthermore, at the serum level, a decrease in molecules involved in the inhibition of the WNT-mediated pathway, such as Dickkopf-related protein (DKK)-1 and sclerostin, was found. Studies carried out at the gene level have also highlighted mutations in key genes of the canonical Wnt/β-catenin pathway, including LRP5, FRZB, and adiponectin (ADIPOQ), a regulator of the Wnt/β-catenin pathway whose anti-inflammatory effects are known [66]; correlated with this discussion is the observation that, in recent years, increasing evidence has been found attesting to the cross-talking between Notch and Wnt signals during organ regeneration [67], and this could suggest the possibility of a defective dialogue between Wnt and Notch underlying the pSjD onset and/or pathogenesis. Similarly, the signal transduction pathway mediated by Hippo activation appears to be involved in the development of SGs, and this was expected given the involvement of Hippo in organ development and cell differentiation and proliferation [68]. In pSjD, an anomalous activation of this pathway occurs, which has, as its consequences, an alteration of cellular integrity and E-cadherin-mediated cell polarity; this leads to the loss of cellular functionality rather than promoting correct cellular interactions. This occurs through the activation of downstream effectors such as TAZ, which localize in the nucleus and act as transcriptional factors due to the inhibition of the upstream kinase Lats2 [69]. This mislocalization results in the accumulation of ECM components, followed by tissue remodeling and fibrosis. Important results obtained from experimental procedures report that the structural defect caused by Hippo signaling dysfunction could be correlated to hyposalivation observed in pSjD, also in an independent way from SGs lymphocytic infiltration. Since defective Notch signaling and Hippo signaling are both linked to NF-κB activation, a probable correlation between the pathways involving the activation of Notch and Hippo and the consequent NF-κB activation could be hypothesized to underlie the pSjD pathogenesis [70,71]. In fact, given that NF-κB and NOTCH pathways are known to interact, it is possible that dysregulated NF-κB signaling may lead to dysregulated Notch signaling, correlated to the Hippo-mediated transductional cascade activation. This hypothesis, however, although very probable, requires experimental confirmation.

The alteration of the structure of the SGs observed in pSjD could also depend on altered communication mechanisms between glandular epithelial cells and stromal cells, which once again seems to involve Notch activation. In fact, the expression of specific genes in stromal cells is regulated by molecules released by epithelial cells in pSjD patients; notably, the release of molecules related to the Notch signaling pathway, such as Notch2/3, appears to depend on epithelial-derived MDK and SCGB3A1, which have the role of promoting transformation into fibroblasts and generate fibrosis [72].

**Figure 4:**
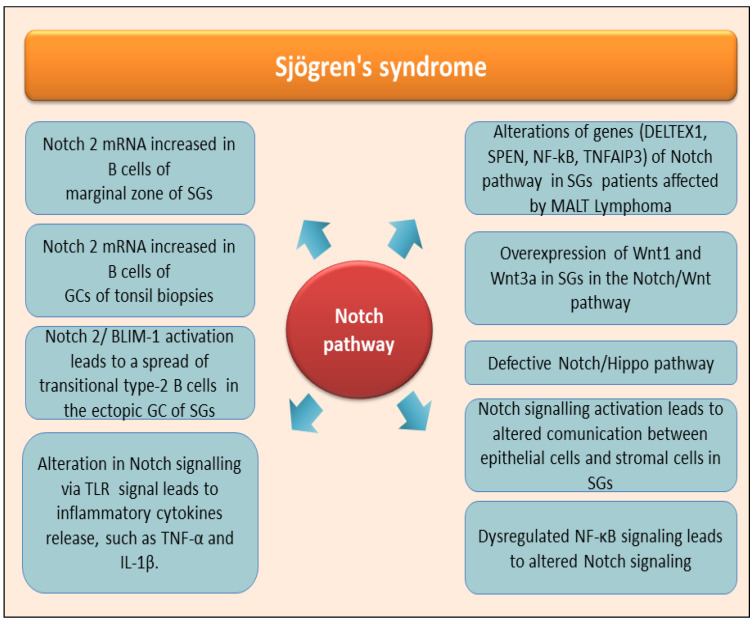
The role of Notch in the Sjögren’s syndrome salivary gland. The patients affected by Sjögren’s syndrome present an altered tissue organization and reduced functionality, primarily of the salivary glands. Altered Notch signaling pathway drivers, through various mechanisms, listed in the scheme, lead to the inflammatory evolution of the disease towards a chronic condition and to the structural disorganization and atrophy of the salivary glands. BLIM-1 (B-lymphocyte maturation-induced maturation protein 1); DELTEX1 (deltex E3 ubiquitin ligase 1); GC (germinal center); NF-kB (Nuclear factor kappa B); SG (salivary gland); SPEN (Split Ends); TNFAIP3 (tumor necrosis factor-α-induced-protein 3); Wnt (wingless-type).

Altered Notch signaling also appears to be involved in characteristic systemic manifestations, characterized by widespread pain, detected in a subgroup of patients with pSjD, probably through TLR signaling [57]. Through the activation of TLR, the activation of Notch signaling determines the activation and proliferation of macrophages and dendritic cells with the production of inflammatory cytokines, such as TNF-α and IL-1β. The molecular characteristics of the pathway just described fully reflect the role of Notch in pSjD, where it appears to be involved in the development of neuropathic pain; the treatment with a Notch signaling inhibitor in these patients can prevent the development of neuropathic pain, and this seems to confirm an involvement of Notch in the painful manifestations associated with pSjD [73]. Considering the published findings on the role of Notch signaling in SjD, actually, there are no contributions related to secondary SjD (sSjD), although this could be an interesting field of research since the mechanisms underlying various pathologies associated with SjD are common; moreover, many of these cascading mechanisms could lead to an activation of Notch. Figure 4 shows current knowledge of the role played by Notch-mediated molecular activation pathways in pSjD.

## 5. Knowledge Regards Notch Pathways Activation in Non-Autoimmune SGs Diseases

Notch signaling was investigated and was demonstrated to play a role also in non-autoimmune SGs diseases [74,75]. Experimental work that evaluates the role of an alteration of Notch expression in SGs pathologies largely focuses on tumors that frequently affect these glands. This is understandable, given the impact of these pathologies on quality of life and the poor prognosis. However, other SGs pathologies could be investigated in the future to identify Notch-mediated mechanisms underlying apparently different pathologies.

### 5.1. Notch Signaling in Adenoid Cystic Carcinoma of Salivary Glands

AdCC (adenoid cystic carcinoma) is one of the most common SGs malignancies [76,77]. The minor SGs represent the elective site for the establishment of these tumors, and the oral cavity is the most frequently affected site. AdCC develops in intercalated ducts, formed internally by ductal epithelial cells and externally by myoepithelial cells. Both cell types play a role in the development of this type of tumor. The histogenesis of AdCC remains unclear, although impaired stem cell development has also been demonstrated. Three histological subtypes of AdCC have been identified: solid, cribriform, and tubular [78]. Carrying out a classification within these pathologies is difficult, as a tumor can present differences that lead to the identification of various subtypes. We distinguish tubular and cribriform tumors and others that progress towards the solid phenotype, determined by the loss of myoepithelial cells and characterized by greater aggressiveness. Therefore, myoepithelial cells are attributed the role of slowing down the evolution towards aggressive forms of solid AdCC [79]. Current knowledge considers myoepithelial cells as cells that rarely undergo transformation and, generally, characterize low-grade aggressive tumors. For example, in breast cancer, myoepithelial cells have been labeled “natural tumor suppressors” due to their negative effects on tumor growth, metastasis, and neoangiogenesis. Their role is carried out thanks to the secretion of protease inhibitors and the downregulation of matrix metalloproteinase levels [80]. Based on these characteristics observed for other tumor forms, the best treatment for primary AdCC is complete surgical excision; however, the role of systemic therapy for AdCC at an advanced stage remains empirical and poorly defined [76,81].

The recent discovery of a high expression of several members of the Notch-mediated molecular activation pathway in the AdCC of SGs fits well within this range of knowledge, comparing them with the expression levels observed in healthy glandular tissues [82]. The interesting data that emerged from the studies concerned a specific correlation between the simultaneous expression of Jagged 1 and Notch 2 and the greater chance of survival in patients with AdCC of the SGs. In particular, it was observed that the presence of both Jagged 1 and Notch2 markers was significantly correlated, from a statistical point of view, with greater chances of survival, while lower chances of survival were correlated with the presence of only one of the markers. It was then hypothesized that, in a cellular environment that expresses the Jagged-1 and Notch-2 ligand together, a phenomenon of tumor growth suppression could occur. This effect is lost when Jagged activates other members of the Notch family (such as Notch1 and Notch4), which leads to the development of more aggressive tumor forms. This has led to the consideration that the expression of Jagged-1, in the absence of the simultaneous expression of Notch-2, has little predictive significance on tumor evolution [82,83]. Therefore, the co-expression of Jagged-1 and Notch-2 appears to be a parameter to monitor to evaluate the survival prospects of patients with human salivary AdCC [82].

### 5.2. The Intriguing Role of Tumor Stem Cells in NOTCH Signaling in Salivary Glands AdCC

An even more intriguing and innovative hypothesis on the role played by Notch in AdCC starts from the analysis of tumor stem cells (CSCs), which represent a subset of tumor cells characterized by self-sufficiency, differentiation capacity, self-renewal, and cellular homeostatic control [84]. Stem cells have been well analyzed and characterized in hematopoietic neoplasms, but specific CSCs in solid tumors still present many unknowns relating to their mechanisms of activation and differentiation. They have, however, been identified in solid tumors of various organs [85]. Based on the known characteristics of tumor stem cells, anti-tumor strategies have been developed based on the inactivation of signals at critical points of the transduction cascades of activation of pathways involved in the proliferation or differentiation of these cells; to this end, several targets have been identified, including Notch, Wnt, and Hedgehog [86,87,88]. Alterations in the regulation of Notch signaling pathways have been studied, predominantly, in breast cancer, demonstrating, both in vitro and in vivo, that the use of gamma-secretase inhibitors that interfere with Notch activation can induce the death of breast cancer cell lines [89]. As Notch signaling has been implicated in CSCs self-renewal, based on the preliminary data obtained, it seems plausible that these gamma-secretase inhibitors may act both on bulk tumor cells but also on CSCs, and this could broaden the prospects of therapeutic success [90]. These inhibitors are currently being tested as anti-tumor drugs [91].

Relating to SGs tumors, accumulating evidence supports the involvement of the CSCs hypothesis in AdCC [92,93]. A recent study reports the identification of cells with biological characteristics similar to CSCs in salivary AdCC; furthermore, scholars have observed that a stabilization or even a decrease in the number of CSCs or a slowdown in their replacement is observed through the inhibition of Notch-mediated signaling [94]. In fact, similarly to what is observed in tumors of other organs, an overexpression of various receptors and ligands involved in the Notch activation pathways has been demonstrated in salivary AdCC. Furthermore, in agreement with what is reported above, in the AdCC of SGs a correlation has been demonstrated between the concomitant expression of Jagged-1/Notch-2 and a better survival prospect [82].

The combined use of different technologies such as NGS and immunohistochemistry has made it possible in recent years to identify and classify AdCC patients with activated NOTCH pathway [95]. Activation of the Notch signaling pathway has been identified as a crucial component in AdCC pathogenesis, with a clinical history evolving towards greater aggressiveness and poor prognosis [83,96].

AdCC patients with mutations in genes involved in the activation of the Notch pathway appear to be more predisposed to the development of liver and/or bone metastases [74]. In this context, in the last years, several investigations were made to clarify the interplay between epithelial-to-mesenchymal transition (EMT) and Notch signaling in the cancer cells dissemination through the blood vessels [83,96,97]. However, currently, it remains in doubt whether Notch has a role in promoting metastasis through the activation of an EMT program.

### 5.3. Notch Signaling in Mucoepidermoid Carcinoma of Salivary Glands

In the field of SGs malignancies, mucoepidermoid carcinoma (MEC) is most common and can also develop in multiple other organs [98]. This type of SGs tumor is histologically characterized by the simultaneous presence of epidermoid cells, mucin-producing cells, and intermediate cells. When diagnosed early, the treatment of the tumor is essentially surgical; unfortunately, diagnosis in an advanced stage or the presence of relapses does not have a favorable prognosis. Currently, no therapeutic procedure undertaken has given a positive outcome, so there is a lack of targeted therapy.

In cases of MEC, a unique chromosomal translocation t (11;19) (q14–21; p12–13) has frequently been identified, which determines the creation of a CRTC1-MAML2 fusion protein [99]. The CRTC1-MAML2 fusion protein presents at the N-terminal region a fragment that derives from the binding domain of the CREB transcriptional co-activator CRTC1, while the C-terminal portion is constituted by the transcriptional activation domain of the Notch transcriptional co-activator MAML2 [99]. This fusion protein is responsible for the neoplastic transformation of epithelial cells. Gene silencing experiments have shown that by inhibiting the transcription of this protein, tumor cells undergo cell death and show a slowdown in tumor growth. This supported the idea that the CRTC1-MAML2 fusion protein represents an oncogenic driver in the development and metastatic evolution of MEC [100]. Based on these findings, targeting the CRTC1-MAML2 fusion could have a potential therapeutic valence. However, the difficulty encountered in designing a therapeutic approach that inhibits CRTC1-MAML2 lies in the fact that this protein is localized in the nucleus and its direct enzymatic activity is not known; for this reason, scholars have focused on identifying signal transduction pathways downstream of the CRTC1-MAML2 fusion in order to identify factors that could be potential therapeutic targets. An interesting result was to demonstrate that CRTC1-MAML2 upregulates the expression of amphiregulin (AREG), an EGFR ligand, thus leading to the activation of transduction cascades in which EGFR activation is involved in an autocrine manner [101]. It has been observed that cell proliferation of human ECMs expressing the fusion protein is sensitive to interference involving EGFR-mediated pathways. Currently, however, a clear involvement of Notch in the regulation of MEC has not been established. Through experiments using dominant negatives for MAML1 or blocking the transcription of the Notch receptor with γ-secretase inhibitors, they have highlighted that Notch appears to be involved in the transformation and proliferation of a small subset of ECM stem cells responsible for tumor metastasis or in the formation of relapses and could therefore be a therapeutic target, but convincing results remain far from being obtained, at least at the current state of research [102].

## 6. Notch-Targeted Therapies in SGs Diseases

The studies reported in the previous paragraphs have made it possible to delve deeper into the possibility of using Notch inhibitors in clinical practice in recent years [103,104]. Experimental evidence has given rise to the awareness that γ-secretase inhibitors, by preventing the release of the active intracellular Notch fragment, represent the elective molecules to be used as Notch inhibitors [89,104,105]. Clinical tests on Notch inhibitors have, however, highlighted intestinal toxicity resulting in diarrhea; therefore, new approaches have been developed in dosing, carried out in an intermittent manner, or the possibility of associating Notch inhibitors with other anti-neoplastic drugs has been evaluated in such a way as to reduce the concentration of Notch inhibitor and alleviate toxicity while maintaining anti-tumor efficacy. A γ-secretase inhibitor, AL101, has also been identified, which acts by inhibiting the Notch activation pathway by interfering with the process of cleavage of the intracellular domain. Also, in this case, the most common problems encountered after administration of the drug were represented by minor intestinal intolerance [106,107]. The γ-secretase inhibitor LY3039478 (trade name Crenigacestat) has demonstrated some clinical efficacy, especially in patients who had undergone previous treatments and were in an advanced stage of the disease or had metastases [108]. In patients with AdCC, crenigacestat showed, however, limited clinical efficacy without the possibility of obtaining certain data [109]. Recently, an increase in therapeutic value has been demonstrated in the case of ECM of SGs if two signal transduction pathways are simultaneously targeted: the one mediated by Notch, which acts on the differentiation and replication of stem cells, and the second is represented by the pathway mediated by the activation of EGFR via the oncogenic fusion protein CRTC1-MAML2 [102,110]. Effective anti-tumor responses were obtained following the simultaneous use of the Notch inhibitor GSI (DBZ) and the EGFR inhibitor (erlotinib); optimal results were obtained by lowering the dosage of both inhibitors but using them at the same time [102]. The data obtained supported the hypothesis that the signaling pathways mediated by Notch and EGFR often interact in a cooperative or antagonistic way depending on the cellular or tissue context in which they are located [111]. The analysis of the complex interdependence between the two activation pathways in human tumors has led to demonstrating how, for example, by blocking the EGFR-dependent pathways, a marked proliferation of tumor stem cells is observed in lung cancer, which occurs precisely following the activation of pathways dependent on Notch activation [112], and simultaneously targeting these two pathways leads to a strong anti-tumor efficacy [113]. In the wake of these experiments, the combined treatment using anti-Notch molecules and anti-EGRF molecules was evaluated in patients in whom tyrosine kinase inhibitors were ineffective. Using drugs such as gefitinib or osimertinib, promising results were obtained in cases of lung cancer [114]. Although there is, therefore, various evidence that Notch and EGFR represent interconnected targets in tumor development, it is currently necessary to investigate further to clarify how EGFR inhibition can influence the signaling pathways triggered by Notch in the ECM, especially at a molecular level where the data collected are few and uncertain. As already reported, the development of AdCC seems to be correlated with mutations in the genes of the factors belonging to the Notch family, and in particular Notch1; a large percentage of patients with AdCC present these mutations, which, in fact, characterize the disease at a more advanced stage. The advanced state is, unfortunately, correlated with the presence of bone and liver metastases and a poor prognosis [74]. For this reason, Notch1 inhibition represents an attractive therapeutic target, and numerous clinical trials are currently underway that rely on the use of Notch1 inhibitors, including brontictuzumab [96] and CB-103, which demonstrated a promising arrest of disease progression [115]. A phase I clinical study tested, in AdCC, the efficacy of brontictuzumab (the molecule is indicated as OMP-52M51), a human-specific monoclonal antibody directed against the Notch1 protein, revealing the effectiveness of this molecule, which is able to repress the proliferation of tumor cells, block the EMT process, often responsible for metastasis, and promote ferroptosis, revealing an enormous therapeutic potential of this antibody [96]. Another more advanced phase II study is, however, evaluating the effectiveness of an inhibitor of the kinases that are activated in stem cells, a drug called amcasertib (BBI-553) in AdCC; the use of this molecule determines the death of tumor stem cells, whose survival is closely related to the activation pathways mediated by Notch, suggesting that the use of this drug interferes with these pathways [116]. Although the structure of the classical transduction cascade involving Notch activation may seem apparently simple, the biological activities in which this cascade is involved are multiple and depend on the cellular environment analyzed and on cell–cell interactions. This has led to the impossibility, up to now, of identifying effective targeted therapies. Table 1 summarizes the molecules used as inhibitors of the transductional activation pathways that involve Notch activation and which have shown some therapeutic efficacy.

## 7. Conclusions

In this review, we have summarized published studies that highlight the role of the evolutionarily conserved Notch signaling pathway in regulating the development of SGs, starting from the discussion of the canonical and non-canonical mechanisms through which the activation of the Notch signaling pathway occurs. In addition, we have provided an overview of the immune regulatory mechanisms of the Notch signaling pathway in autoimmune and non-autoimmune SGs diseases, highlighting various molecular gaps common to all the pathologies examined.

The data collected highlight the importance of a crosstalk between Notch and other signaling and of identifying molecular bridges that can connect the signal transduction pathways that converge on Notch. Unfortunately, no single clinical drug to date has shown enough efficacy and safety to be used in diseases that have shown an alteration of the Notch signal. This raises the need to discover new strategies that can lead to the identification of an effective drug in Notch-mediated autoimmune and non-autoimmune diseases such as pSjD or tumors. The key role of Notch signaling as a promising therapeutic target in SGs diseases is confirmed by the contribution given by this molecule in the development of SGs, which could explain how its altered gene or protein expression could be at the basis of the developed pathological forms. Therefore, future studies must be aimed at clarifying the balance between the positive and negative effects that derive from Notch activation.

There is currently a lot of research on the possibility of using Notch signaling pathways to identify targeted therapies. Two possible lines of investigation have been identified to this end, both based on the possibility of blocking Notch-mediated signal transduction pathways. The first approach followed was based on the possibility of identifying inhibitors that at various levels can block crucial molecules of the pathway, such as the enzymes involved in the cleavage of S1, ADAM, γ-secretase enzymes, and MAML. The second approach was aimed at designing drug-antibody fusion molecules that would act by directly blocking Notch receptors and ligands. Few promising results, however, have been obtained. Overall, researchers agree in considering the Notch signaling pathway as a potential target for immunomodulatory therapies in autoimmune, inflammatory, or tumor diseases. Given the close link between chronic inflammation and autoimmune diseases, and, considering that chronic inflammation may underlie cellular alterations that lead to the development of neoplasia, studies should be directed to co-validate the use of Notch inhibitors to reduce tissue inflammation and minimize tissue damage. Unfortunately, this remains a significant open challenge. Further research is needed to clarify the mechanism of various Notch receptors and ligands in pSjD, to assess whether Notch activation pathways are relevant to other salivary gland diseases, which is currently an uninvestigated field, and to clarify the role of Notch in SGs tumors. For this reason, we believe that this review can guide and channel future studies by exploiting the knowledge acquired in the pathological field in the salivary glands. They may seem like niche experiments, but the latest discoveries have identified multiple mechanisms common to various inflammatory and tumor pathologies, applicable to any other experimental model with the aim of freeing and promoting new insights in this field.

## Figures and Tables

**Table 1:** Drugs targeting the Notch signaling pathway.

Drug	Target	Mode of Action	Effect	Ref
AL101	γ-secretase inhibitor	Notch pathway inhibition during the cleavage process in the intracellular domain	Anti-tumor effect in patients with metastatic solid tumors	[106]
Crenigacestat	γ-secretase inhibitor	Inhibition of the release of the Notch intracellular domain by suppression of γ-secretase complex	Anti-tumor effect in patients with advanced or metastatic disease as patients with ADCC	[108]
DBZ dibenzazepine	γ-secretase inhibitor	The block of the cleavage of Notch into its active signaling effector, Notch intracellular domain	Anti-tumor efficacy with strong anti-stem cell effect in human mucoepidermoid carcinoma of the salivary gland	[102]
Erlotinib	EGFR inhibitor	Inhibition of the intracellular phosphorylation of tyrosine kinase	Anti-tumor effect in human mucoepidermoid carcinoma	[102]
Gefitinib	EGFR inhibitor	Inhibition of the intracellular phosphorylation of tyrosine kinase	Anti-tumor effect in lung adenocarcinoma	[114]
Osimertinib	EGFR inhibitor	Inhibition of the intracellular phosphorylation of tyrosine kinase	Anti-tumor effect in lung adenocarcinoma	[114]
Brontictuzumab	Notch 1 inhibitor	Humanized monoclonal antibody against the Notch1 protein	Repression of the proliferation and EMT inhibiting salivary adenoid cystic carcinoma	[96]
CB-103	Notch 1 inhibitor	Inhibits the CSL–NICD	Downregulation in Notch signaling	[115]
Amcasertib	Notch inhibitor	Cancer stemness kinase inhibitor	Impairment of cancer stem cell survival deregulating Notch signaling in AdCC	[116]
